# Leptin regulates CD16 expression on human monocytes in a sex‐specific manner

**DOI:** 10.14814/phy2.12177

**Published:** 2014-10-10

**Authors:** Joseph G. Cannon, Gyanendra Sharma, Gloria Sloan, Christiana Dimitropoulou, R. Randall Baker, Andrew Mazzoli, Barbara Kraj, Anthony Mulloy, Miriam Cortez‐Cooper

**Affiliations:** 1 College of Allied Health Sciences, Georgia Regents University, Augusta, Georgia; 2 Medical College of Georgia, Georgia Regents University, Augusta, Georgia

**Keywords:** Blood pressure, carotid compliance, intima‐media thickness, leptin receptor

## Abstract

Fat mass is linked mechanistically to the cardiovascular system through leptin, a 16 kDa protein produced primarily by adipocytes. In addition to increasing blood pressure via hypothalamic‐sympathetic pathways, leptin stimulates monocyte migration, cytokine secretion, and other functions that contribute to atherosclerotic plaque development. These functions are also characteristics of CD16‐positive monocytes that have been implicated in the clinical progression of atherosclerosis. This investigation sought to determine if leptin promoted the development of such CD16‐positive monocytes. Cells from 45 healthy men and women with age ranging from 20 to 59 years were analyzed. Circulating numbers of CD14^++^16^++^ monocytes, which are primary producers of TNFα, were positively related to plasma leptin concentrations (*P* < 0.0001), with a stronger correlation in men (*P* < 0.05 for leptin × sex interaction). In vitro, recombinant human leptin induced CD16 expression in a dose‐related manner (*P* = 0.02), with a stronger influence on monocytes from men (*P* = 0.03 for leptin × sex interaction). There were no sex‐related differences in total leptin receptor expression on any monocyte subtypes, relative expression of long versus short isoforms of the receptor, or soluble leptin receptor concentrations in the plasma. The number of circulating CD14^+^16^++^ monocytes, which preferentially migrate into nascent plaques, was positively related to systolic blood pressure (*R* = 0.56, *P* = 0.0008) and intima‐media thickness (*R* = 0.37, *P* = 0.03), and negatively related to carotid compliance (*R* = −0.39, *P* = 0.02). These observations indicate that leptin promotes the development of CD16‐positive monocyte populations in a sex‐specific manner and that these subpopulations are associated with diminished vascular function.

## Abstract (precis)

e12177

Recombinant leptin induced CD16 expression on human monocytes in vitro in a dose‐ and sex‐specific manner. In vivo, CD16 expression on human monocytes correlated with plasma leptin concentrations in a sex‐specific manner. Blood pressure, carotid intima‐media thickness and carotid compliance were related to the number of circulating CD16‐positive monocytes.

## Introduction

Cardiovascular disease affects one‐third of the United States population, and obesity‐related metabolic disorders are considered to be major causes (Begg et al. 2006). One mechanistic link between body fat mass and the cardiovascular system is leptin, a 16 kDa protein produced primarily by adipocytes. Leptin was originally identified as a regulator of food intake and energy expenditure (Correia and Haynes 2004), but it also increases blood pressure (Hall et al. 2010) and promotes the development of atherosclerosis (Beltowski 2006).

The hypertensive action of leptin is mediated via receptors in the arcuate nucleus and other regions of the hypothalamus that activate pro‐opiomelanocortin neurons and subsequently upregulate the sympathetic nervous system (do Carmo et al. 2011). The slowly developing increase in blood pressure is thought to be due primarily to increased renal sympathetic nerve activity and subsequent sodium retention (Hall et al. 2010). Numerous human studies have reported positive correlations between circulating leptin concentrations and blood pressure after controlling for body fat (Kennedy et al. 1997; Schorr et al. 1998; Suter et al. 1998; Kazumi et al. 1999; Guagnano et al. 2003).

Leptin receptors are also expressed on blood monocytes (Zarkesh‐Esfahani et al. 2001), and leptin promotes the development of monocyte activities that can contribute to the progression of atherosclerosis. These include enhanced adhesion and migration through the extracellular matrix, upregulated scavenger receptor expression, increased uptake of oxidized low‐density lipoprotein (LDL) (Konstantinidis et al. 2008), and secretion of TNFα (Santos‐Alvarez et al. 1999; Zarkesh‐Esfahani et al. 2001).

These functional changes suggest that leptin might induce a shift in the monocytes from a CD16‐negative to a CD16‐positive phenotype (Santos‐Alvarez et al. 1999; Zarkesh‐Esfahani et al. 2001). Whereas all circulating monocytes express CD14, which is the receptor for bacterial lipopolysaccharide (LPS), only a small subpopulation (10–15%) coexpresses CD16 (Passlick 1989), which is an FcγIII receptor that binds IgG complexed with modified LDL (Kiener et al. 1995). These CD16‐positive monocytes were first described as a single subpopulation (Passlick 1989) that, compared with CD16‐negative monocytes, were smaller, had somewhat diminished expression of CD14, contained fewer cytosolic granules, and exhibited a lower rate of phagocytosis (Belge et al. 2002; Geissmann et al. 2003). These cells express a different set of chemokine receptors and adhesion molecules, and therefore have different homing characteristics and functional properties than CD16‐negative monocytes (Weber et al. 2000). They also tend to crawl across the luminal surface of endothelial cells in patterns independent of the direction of blood flow in a “patrolling” behavior thought to facilitate rapid extravasation upon encountering local tissue damage (Auffray et al. 2007).

Based on this original classification scheme, CD16‐positive monocytes were associated with cardiovascular disease. Patients with coronary artery disease exhibited higher circulating numbers of CD16‐positive monocytes than healthy controls (Schlitt et al. 2004), and those in the highest quartile for circulating CD16‐positive monocytes also had significantly higher serum TNFα concentrations. Moreover, CD16‐positive monocytes accumulate in human atherosclerotic lesions (Hakkinen et al. 2000). In vitro, antibodies to CD16 blocked the vascular inflammation caused by IgG‐activated human macrophages (Boyle et al. 2012).

More recent studies have shown that CD16‐positive monocytes can be further subdivided into CD14^++^16^++^ and CD14^+^16^++^ subpopulations that have different functional properties. Whereas CD16‐negative (CD14^++^) monocytes were shown to be the primary producers of IL‐10 and reactive oxygen species (Cros et al. 2010), monocytes that strongly express CD14 along with CD16 (CD14^++^16^+^ and CD14^++^16^++^) are the primary sources of IL‐1β and TNFα. Monocytes that strongly express CD16 but less CD14 (CD14^+^16^++^) exhibit the patrolling behavior leading to extravasation (Cros et al. 2010).

The aim of the present study was to test the hypothesis that leptin induces CD16 expression on monocytes, and to determine if increased CD16 expression is related to carotid intima‐media thickness (IMT), compliance, and blood pressure in generally healthy human subjects.

## Methods

### Subjects

The first phase of the study involved 34 subjects (median age 24 years), including 13 men, 11 women with natural menstrual cycles, and 10 women who were using oral contraceptives. Exclusion criteria included smoking, current pregnancy or lactation, or a history of chronic diseases such as diabetes, liver disease, cardiovascular disease or cancer, or any use of prescription drugs in the past 3 months.

Subjects reported for a first visit to the laboratory between 7:30 and 9:00 am, having fasted for at least 8 h, and refrained from vigorous physical activity the preceding day. All women were tested in the follicular phase of their menstrual cycles (or the placebo phase of oral contraceptive cycles). Information regarding general health was collected via questionnaire and self‐reported physical activity was quantified using the Godin and Shephard (1985) scale. This was followed by noninvasive vascular testing and lastly, acquisition of a blood sample by venipuncture of the antecubital vein. On a second visit, usually separated from the first by less than 2 weeks, the aerobic capacity and body composition of the subjects were determined.

In the second (follow‐up) phase designed to determine the causal influence of leptin on monocyte expression of CD16, blood samples from an additional six men and five women (four on oral contraceptives, median age 25 years) were collected for mononuclear cell isolation and incubation with recombinant leptin in vitro. The women were tested in the follicular or placebo phases of their cycles. All procedures were approved by the Human Assurance Committee (the Investigation Review Board) at the Georgia Regents University, and all subjects provided written informed consent.

### Blood analyses

#### Blood collection and leukocyte isolation

Blood was collected into a 7 mL untreated tube for serum and a 6 mL EDTA‐treated tube for plasma. The serum and plasma were aliquoted and frozen at −70°C for later analysis. In addition, three 10 mL sodium heparin‐treated tubes were drawn and the mononuclear cells were isolated by density gradient centrifugation for cell culture.

#### In vitro cytokine secretion

Mononuclear cells were isolated by density gradient centrifugation using Ficoll‐Hypaque (Sigma, St. Louis, MO), and resuspended in RPMI‐1640 medium (free of phenol‐red to avoid estrogen‐like influences) supplemented with 2 mmol/L of l‐glutamine, 100 U/mL penicillin, 100 *μ*g/mL streptomycin, 25 mmol/L Hepes buffer (all from Mediatech, Manassas, VA), and 1% heat‐inactivated autologous plasma. Cells were then distributed in 24‐well polystyrene plates (Corning Glass Works, Corning, NY) at 2.5 × 10^6^ cells/well and incubated with 0, 50, or 100 ng/mL of recombinant human leptin (Cat # 398‐LP, R&D Systems, Minneapolis, MN, <1 endotoxin unit/*μ*g protein) for 18 h at 37°C in a humidified 5% CO_2_ atmosphere. After incubation, cell supernatants were collected and centrifuged at 12,000 *g* for 1 min to remove any aspirated cells, then aliquoted and frozen at −70°C for later cytokine analysis.

#### Cytokine analyses

Plasma and supernatant concentrations of IL‐1*β*, TNF*α*, IL‐6, and serum concentrations of leptin were measured using a cytometric bead array multiplex methodology (CBA, BD Biosciences, San Jose, CA) and analyzed with a BD FACSArray. The assays had detection limits (based on 95% confidence intervals over blank) of <5 pg/mL. The intra‐assay coefficients of variability (COVs) were between 4% and 10%; the interassay COVs were between 4% and 15%. Serum soluble leptin receptor concentrations were determined with an ELISA kit from R&D Systems. The detection limit was 0.06 ng/mL, inter‐ and intra‐assay COVs were 8.6% and 6.1%, respectively.

#### Hormone assays

Serum estradiol, progesterone, testosterone, and cortisol concentrations were measured with ELISA kits from Bio‐Quant (San Diego, CA) with intra‐assay COVs between 6% and 9%, and interassay COVs between 9% and 15%. Plasma insulin and serum oxidized LDL concentrations were determined with ELISA kits from ALPCO (Salem, NH). The intra‐assay COVs were 3–10% and the interassay COVs were 7–17%.

#### Glucose and lipoprotein assays

Serum glucose, triglyceride, total cholesterol, and high‐density lipoprotein/cholesterol (HDL) concentrations were measured in a CLIA‐certified core laboratory on the GRU campus using an automated procedure (Envoy 500; Vital Diagnostics Inc., Lincoln, RI).

#### Flow cytometric analysis of cell surface proteins

**Figure 1.**
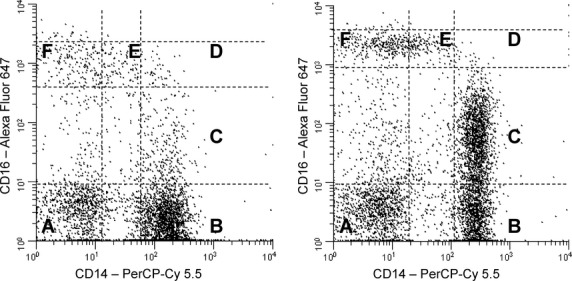
Two representative examples of CD14 and CD16 expression on monocytes (after forward‐/side‐scatter gating). CD14^++^ monocytes are contained in region B, CD14^++^16^+^ monocytes are in region C, CD14^++^16^++^ monocytes are in region D, and CD14^+^16^++^ monocytes are in region E. Region A contains cells that express neither CD14 nor CD16; region F contains cells that are CD16‐positive but CD14‐negative. Regions A and F were used for defining the other regions, as described in the Methods section.

After removal of plasma from centrifuged EDTA‐treated blood, cells were washed in PBS without Ca^++^ or Mg^++^, then labeled with monoclonal antibodies directed against CD14 (clone M5E2, PerCP‐Cy 5.5 label), CD16 (clone 3G8, Alexa Fluor 647 label) (both from BD Biosciences, San Jose, CA), and leptin receptor (clone 52263, phycoerythrin [PE]‐label, R&D Systems). Two additional incubation conditions were set up with antibodies against CCR2 (clone 48607, PE label, R&D Systems) and CX3CR1 (clone 2A9‐1, PE label, BioLegend, San Diego, CA) in place of antileptin receptor. After 30 min, PharmLyse (BD Biosciences) was added to each tube, and incubated in the dark at room temperature for 20 min to lyse the red cells. The remaining leukocytes were centrifuged, washed, treated with stabilizing fixative (BD Biosciences), filtered through 35 *μ*m mesh, and analyzed using a Becton Dickinson 2‐laser, 4‐detector FACSCalibur Flow Cytometer calibrated with Calibrite beads. For each sample, 5000 events were counted in the monocyte gate identified by a dual parameter plot of forward scatter versus side scatter. In the later studies with the second cohort of subjects (in vitro induction of CD16 by leptin), monocytes were gated by CD86 versus side scatter (using anti‐CD86 clone 2331 [FUN‐1] from BD Bioscience). Multicolor flow cytometry was performed with appropriate one‐color controls for setting compensation for spectral overlap. Isotype controls were run to test for nonspecific binding. Figure 1 illustrates how CD14/CD16 expression was categorized. Strict rectilinear regions with vertical boundaries defined by the bimodal distribution for CD14 in CD16‐negative cells (regions A and B), and horizontal boundaries defined by the bimodal distribution of CD16 in CD14‐negative cells (regions A and F) were constructed for each dot plot.

#### Western blot analysis of leptin receptor isoforms

Cells were isolated from heparinized blood by density gradient centrifugation as described previously, washed in sterile saline, and then 10^6^ cells were lysed in 200 *μ*L of Laemmli sample buffer (Bio‐Rad, Hercules, CA) heated to 95°C for 5 min. Lysates were frozen at −70°C until analyzed. Samples were electrophoresed on a 4–20% TGX Criterion gel (Bio‐Rad) at 200 V constant voltage, then transferred to a polyvinylidene difluoride (PVDF) membrane at a constant voltage of 100 V. Membranes were washed in PBS/Tween 20, blocked in 5% milk, then labeled with monoclonal antileptin receptor (clone 52263, R&D Systems), and a secondary anti‐mouse IgG conjugated to horseradish peroxidase (Jackson ImmunoResearch Laboratories, West Grove, PA). The labeled membrane was stained using West Femto chemiluminescent substrate (ThermoScientific, Rockford, IL), imaged using a ProteinSimple FluorChem Imager (ProteinSimple, Santa Clara, CA), and analyzed with AlphaView software (ProteinSimple).

#### Leptin‐induced CD16 expression in vitro

In early experiments, cells were incubated in polypropylene tubes under the conditions described previously for cytokine secretion, then analyzed for membrane expression of CD14 and CD16 by flow cytometry. These preliminary experiments yielded no evidence of an influence by leptin and were discontinued due to resource/logistical limitations. However, after an in vivo association between serum leptin and native CD16 expression became evident, blood samples from 11 additional subjects were collected and their mononuclear cells were isolated and incubated as in the earlier experiments, except higher concentrations of leptin were used: 10 and 100 nmol/L (160 and 1600 ng/mL). After 18 h of incubation, the cells were analyzed for membrane expression of CD14 and CD16 by flow cytometry.

### Vascular testing

All vascular measures were measured twice during a given testing session and averaged.

#### Blood pressure

Seated and supine blood pressures were measured via automatic oscillometry (Dinamap ProCare 100, General Electric) in the left arm after at least 5 min of rest. Supine brachial blood pressure was taken periodically during the vascular testing procedures and was used to calculate carotid compliance.

#### Carotid arterial compliance

Arterial compliance of the carotid artery was determined by the technique of ultrasound imaging with sequential applanation tonometry. Briefly, the common carotid artery diameter was measured from images derived from an ultrasound machine (Envisor, Philips Medical, Bothel, WA) equipped with a high‐resolution, 7.5 MHz linear probe (Cortez‐Cooper et al. 2008). These ultrasound images were transferred to a computer, digitized and analyzed for maximal systolic expansion, and basal (minimum) diastolic relaxation using semiautomated edge‐detection image analyzer software (Carotid artery analyzer tool, Medical Imaging Applications, LLC, Coralville, IA). All image analyses were performed by the same investigator to eliminate intertester variability.

Common carotid blood pressure waveforms were obtained from the ipsilateral artery with a pencil‐type probe incorporating a pressure transducer (SPT 301, Millar Instruments, Houston, TX) and interfaced with a computer (PowerLab, ADInstruments, Colorado Springs, CO). Carotid systolic blood pressure was corrected for hold down pressure as described by Armentano et al. (1995). A minimum of 20 cardiac cycles were captured for offline analysis.

Arterial compliance and carotid pulse pressure were calculated as described previously Cortez‐Cooper et al. (2008). In our laboratory, the mean difference for test–retest reliability for carotid pulse pressure is 1.5 ± 1.6 mmHg and the ICC_1,1_ is 0.85. For carotid end‐diastolic diameter, ICC_1,1_ is 0.93 with a mean difference of 0.21 ± 0.12 mm.

#### Carotid IMT

Carotid IMT was measured from the ultrasound images recorded during compliance measurements. IMT was measured at end‐diastole with the lumen‐intima and media‐adventitia boundaries traced automatically and calculated as the average of 0.1 mm samples over 10 mm using specialized software (carotid artery analyzer tool, Medical Imaging Applications, LLC, Coralville, IA).

### Aerobic capacity testing

Aerobic fitness can influence vascular function, therefore we measured aerobic capacity (VO_2_peak) by open‐circuit spirometry (Sensormedics Vmax Spectra, Yorba Linda, CA) during an incremental cycle ergometer exercise protocol. The cycling rate was held constant at 70 rpm and the resistance was increased 25 W every 2 min after an initial 3‐min warm‐up at 25 W. A 12‐lead ECG was monitored continuously, and blood pressure (conventional sphygmomanometry) and ratings of perceived exertion (Borg RPE scale) were recorded at the end of each stage. Exercise continued until subject exhaustion and an RPE of ≥17. All exercise testing was supervised by a cardiologist. Results reported in this manuscript are expressed as milliliters of oxygen consumed per minute per kilogram of fat‐free mass (mL O_2_/min/kg ffm).

### Body composition

Fat‐free soft tissue and percent body fat were determined by dual energy X‐ray absorptiometry whole body scan (Hologic Discovery W Densitometer, Hologic Inc., Bedford, MA). Each scan was compartmentalized and analyzed using Hologic software.

### Data analysis

All statistical analyses were performed using Statview statistical software (SAS, Cary, NC). Circulating CD16‐positive monocyte counts were normalized by square root transformation, whereas serum leptin and oxidized LDL concentrations were normalized by log transformation. Results are reported by mean ± SEM, unless specifically stated otherwise. Differences in surface antigen expression on monocyte subpopulations were assessed by analysis of variance, using the Bonferroni/Dunn test for multiple pairwise post hoc comparisons. Leptin‐induced CD16 expression was assessed by two‐factor (leptin dose × sex) repeated measures ANOVA. Correlations between variables were analyzed by simple regression, multiple regression, or analysis of covariance, as stipulated in the text. Stepwise multiple regression analyses were performed using an *F*‐to‐enter of 4.0 and standardized regression coefficients (*b*) are reported for independent factors. A *P*‐value <0.05 was considered statistically significant.

## Results

### Subject characteristics

The characteristics of the first cohort of research subjects are presented in Table 1. The men were significantly taller and heavier, whereas the women had significantly higher body fat percentages, serum leptin, and HDL concentrations. Seven women and six men were classified as prehypertensive, with casual systolic blood pressures ≥120 mmHg. No significant sex‐related differences were found for age, aerobic capacity (normalized to fat‐free mass), physical activity levels, blood pressure, blood glucose, or other cholesterol concentrations. Serum leptin concentrations correlated positively with % body fat (*R* = 0.91, *P* < 0.0001).

**Table 1.** Physical characteristics and blood analyses of the research subjects.

	Men (*n* = 13)	Women (*n* = 21)	*P*‐value
Age (years)	26.6 (21–36)^1^	26.1 (21–40)^1^	–
Height (meters)	1.76 ± 0.02	1.63 ± 0.01	<0.0001
Weight (kg)	81.8 ± 3.8	67.1 ± 2.4	0.002
Body fat (%)	19.9 ± 1.7	30.5 ± 1.4	<0.0001
Godin score	39.6 ± 7.4	35.0 ± 5.6	–
VO_2_max (mL/min/total kg)	34.2 ± 3.1	28.2 ± 1.6	0.064
VO_2_max (mL/min/fat‐free kg)	45.6 ± 3.4	43.9 ± 1.8	–
Systolic BP (mmHg)	118 ± 3	113 ± 2	–
Diastolic BP (mmHg)	76 ± 3	72 ± 2	–
Glucose (mg/dL)	88.1 ± 1.5	85.7 ± 1.6	–
Insulin (μIU/mL)	6.02 ± 1.11	7.30 ± 0.98	–
Cortisol (ng/mL)	128 ± 29	96 ± 8	–
Total cholesterol (mg/dL)	169 ± 8	162 ± 6	–
HDL (mg/dL)	42.2 ± 2.7	53.1 ± 2.0	0.003
LDL (mg/dL)	105 ± 7	89 ± 7	–
Oxidized LDL (ng/mL)	235 (763)^2^	197 (1735)^2^	–
Triglycerides (mg/dL)	91.3 ± 11.2	70.1 ± 5.7	0.08
Leptin (ng/mL)	4.60 (8.82)^2^	9.79 (9.05)^2^	0.001
Soluble leptin receptor (ng/mL)	25.9 ± 1.7	30.1 ± 2.6	–

All values are mean ± standard error, except as indicated. –: Not statistically significant.

^1^ Mean (range).

^2^ Median (interquartile range).

### Circulating monocyte subpopulations

**Figure 2.**
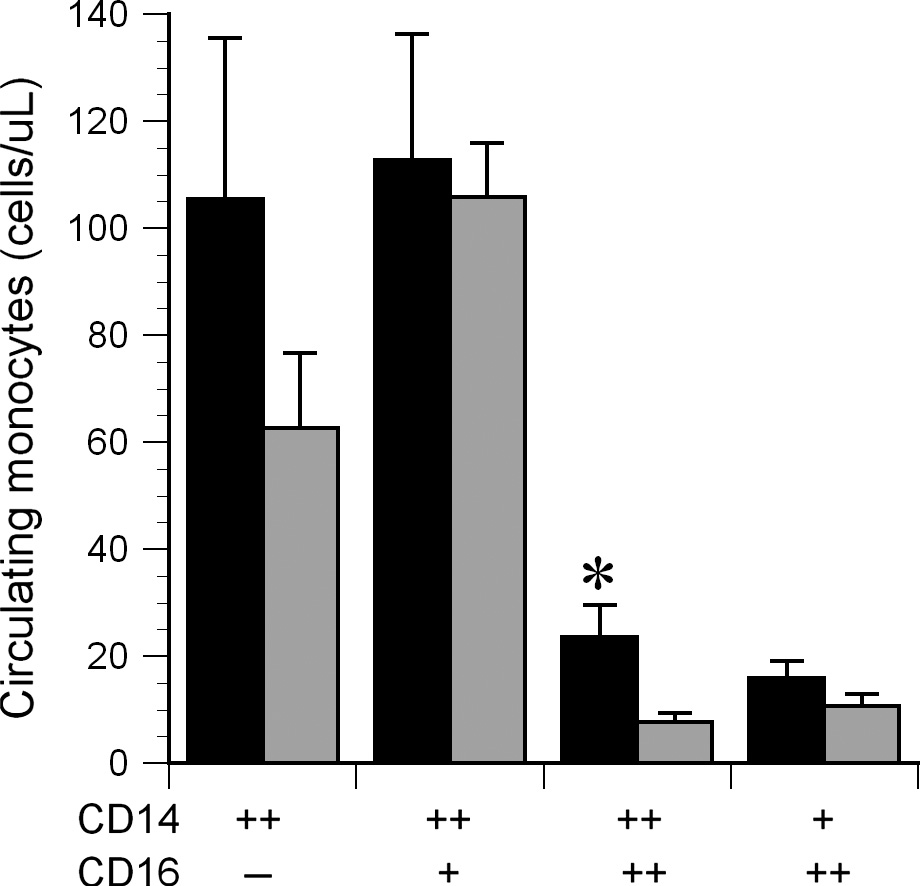
Circulating monocyte counts (*n* = 34). Black bars represent men, gray bars represent women. *The CD14^++^16^++^ monocyte count was significantly higher in men than women, *P* = 0.001.

Two distinct patterns of CD14^++^ monocyte subpopulations were observed among the subjects: either the majority of CD14^++^ cells were CD16‐negative (Fig. 1A) or else expressed low levels of CD16 (CD14^++^16^+^, Fig. 1B). Together, these two subpopulations accounted for 81.4% of total monocytes, whereas 6.3% were CD14^++^16^++^ and 5.7% were CD14^+^16^++^. Overall, circulating monocyte counts were higher in men than in women (*P* = 0.02), although within specific subpopulations the difference was statistically significant only for the CD14^++^16^++^ cells (*P* = 0.001, Fig. 2).

**Figure 3.**
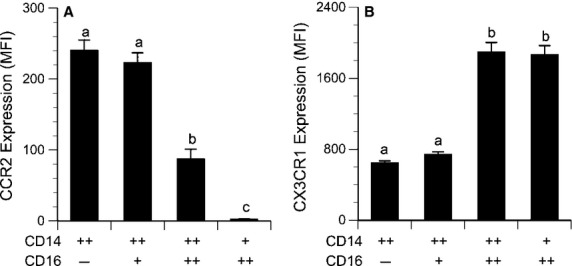
Receptor expression on circulating monocyte subpopulations expressed as mean fluorescence intensity minus fluorescence of the isotype control (*n* = 34). (A) CCR2: *a* > *b* > *c*, all pairwise comparisons *P* < 0.0001. (B) CX3CR1: *a* < *b*,* P* < 0.0001.

The CD14^++^ and CD14^++^16^+^ cells were similar in terms of chemokine receptor expression (Fig. 3) and differed greatly from the CD14^++^16^++^ cells, which expressed less than half as much CCR2 and over twice as much CX3CR1. The CD14^+^16^++^ monocytes expressed essentially no CCR2, whereas CX3CR1 levels were similar to the CD14^++^16^++^ cells.

### Leptin and CD16 expression

**Figure 4.**
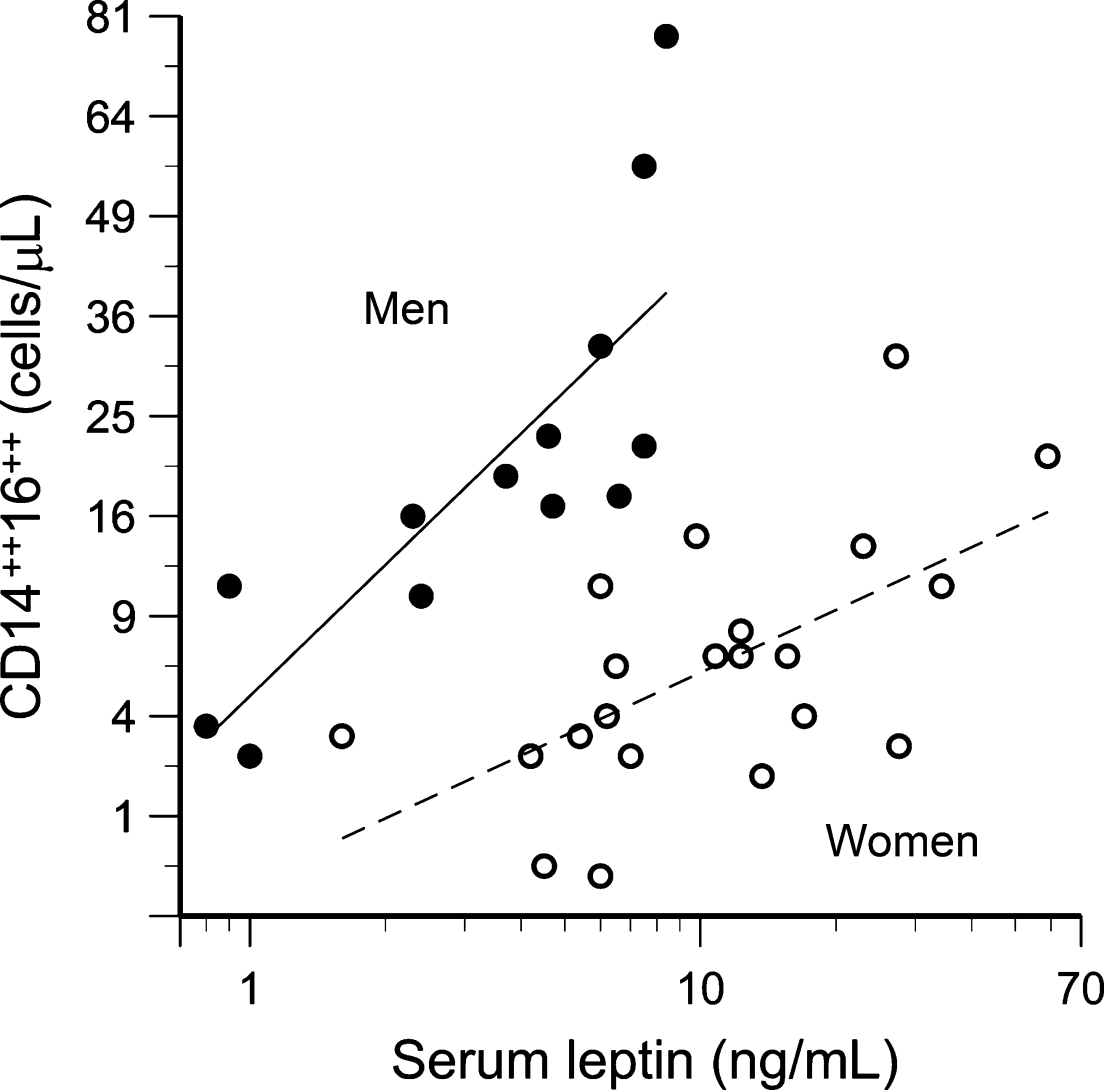
Circulating CD14^++^16^++^ monocytes correlate with serum leptin concentrations (*P* < 0.0001) in a sex‐specific manner (leptin × sex interaction *P* < 0.05). Mean CD14^++^16^++^ counts were significantly higher for men than women (*P* < 0.0001), and exhibited a stronger correlation with serum leptin (*R* = 0.80, *P* = 0.0009) than for women (*R* = 0.60, *P* = 0.005).

**Figure 5.**
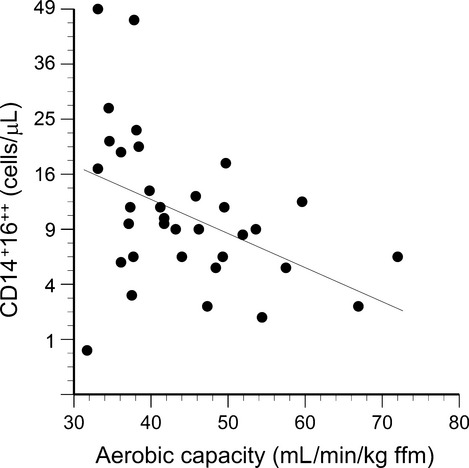
Circulating CD14^+^16^++^ cells plotted as a function of the cell donor's aerobic capacity (*R* = −0.41, *P* = 0.02).

The number of circulating CD16‐positive monocytes was related in a sex‐specific manner to serum leptin concentration, with the strongest association exhibited by the CD14^++^16^++^ cells (Fig. 4). The *P*‐values for leptin correlations with CD14^++^16^+^ cells and CD14^+^16^++^ cells were 0.06 and 0.01, respectively. The CD16‐negative (CD14^++^) cells exhibited no association with serum leptin (*P* = 0.51). Age, body fat, sex hormone concentrations, glucose, and serum lipids were not retained as significant factors when included along with leptin in stepwise multiple regressions for any of the monocyte subpopulations. Aerobic capacity was identified as a significant factor for the CD14^+^16^++^ subpopulation only (Fig. 5).

**Figure 6.**
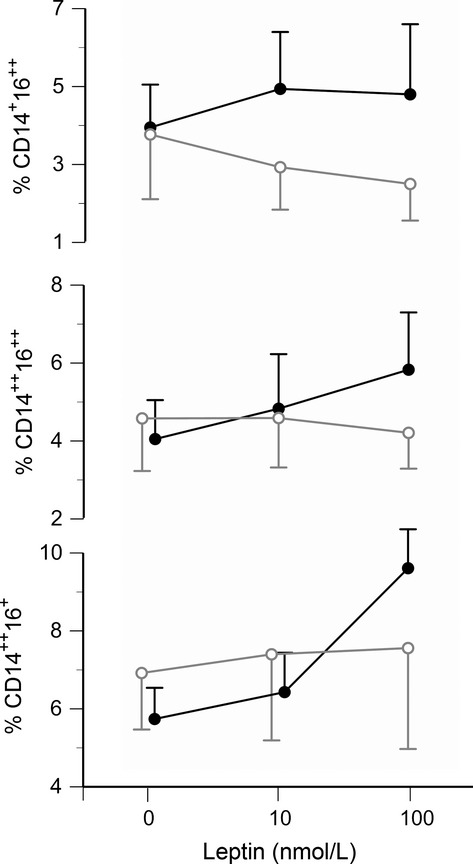
Leptin‐induced expression of CD16 on CD86^+^ monocytes in vitro. The three panels show the percentages of each cell type after 18 h of incubation in vitro with 0, 0.16, or 1.6 *μ*g/mL leptin. The solid black symbols represent cells from men (*n* = 6) and the open gray symbols represent cells from women (*n* = 5). A significant main effect by leptin was observed for CD14^++^CD16^+^ monocytes (bottom panel, *P* = 0.02), and significant leptin × sex interactions were observed for CD14^++^CD16^++^ and CD14^+^CD16^++^ cell populations (middle and top panels, both *P* = 0.03). In the men only, leptin‐induced increases were statistically significant for the CD14^++^16^+^ (*P* = 0.0002) and CD14^++^16^++^ (*P* = 0.004) populations.

Leptin induced sex‐specific increases in CD16 expression in isolated mononuclear cells incubated in vitro with 10 and 100 nmol/L doses of human recombinant leptin. The results are illustrated in Figure 6. A statistically significant main effect of leptin was observed for the CD14^++^16^+^ monocytes (*P* = 0.02), and significant leptin × sex interactions were observed for the CD14^++^16^++^ and CD14^+^16^++^ subpopulations (*P* = 0.03).

### Leptin receptor expression

**Figure 7.**
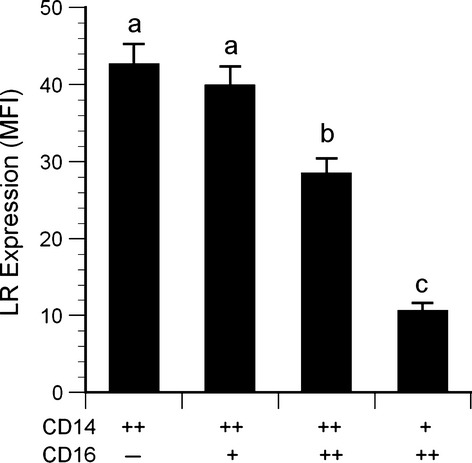
Native leptin receptor expression on monocytes. Data for each monocyte subtype are expressed as median fluorescence intensity minus isotype control fluorescence. (*n* = 34). Statistical key: *a* > *b* > *c*, all pairwise comparisons *P* < 0.002.

**Figure 8.**
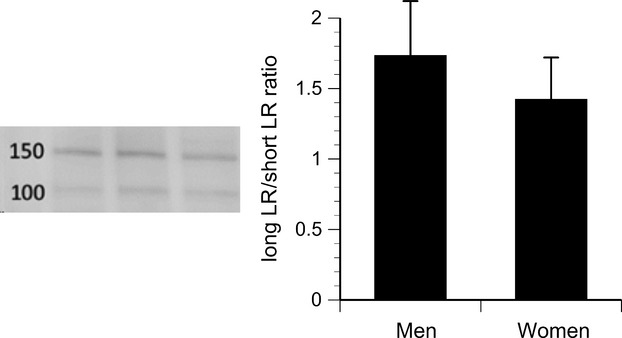
Left: Western blot results for three subjects showing relative expression of long leptin receptor isoform (150 kDa) and short isoforms (100 kDa). Right: Densitometric measurements are presented as the ratio of long/short isoforms, for men versus women. No statistically significant sex difference was observed.

Leptin receptor expression quantified by flow cytometry was highest on CD14^++^ and CD14^++^16^+^ monocytes, intermediate on CD14^++^16^++^ monocytes, and lowest on CD14^+^16^++^ cells (Fig. 7). No sex‐related differences in expression were detected. Western blots revealed two distinct bands at approximately 150 and 100 kDa (Fig. 8), corresponding to the long and short isoforms of the leptin receptor reported previously for human macrophages (Hongo et al. 2008). No sex‐related differences were detected in the relative expression (ratio) of the long/short isoforms quantified by densitometry (Fig. 8), or in circulating concentrations of soluble leptin receptors (Table 1).

**Figure 9.**
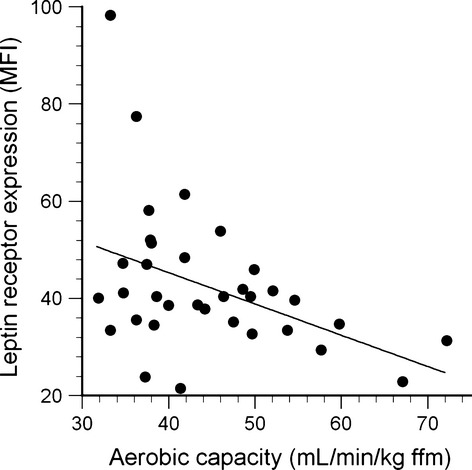
Leptin receptor expression on CD14^++^ monocytes plotted as a function of aerobic capacity (*R* = −0.42, *P* = 0.01).

Leptin receptor expression was inversely related to aerobic capacity for the CD14^++^ subpopulation (*R* = −0.42, *P* = 0.01, Fig. 9) and the CD14^++^16^+^ subpopulation (*R* = −0.35, *P* = 0.04, not shown), but not the CD14^++^16^++^ or CD14^+^16^++^ subpopulations. Age, body fat, aerobic capacity, and traditional cardiovascular risk factors (glucose, lipids) were not retained as significant factors when included in stepwise multiple regressions.

### Leptin‐induced cytokine secretion

**Figure 10.**
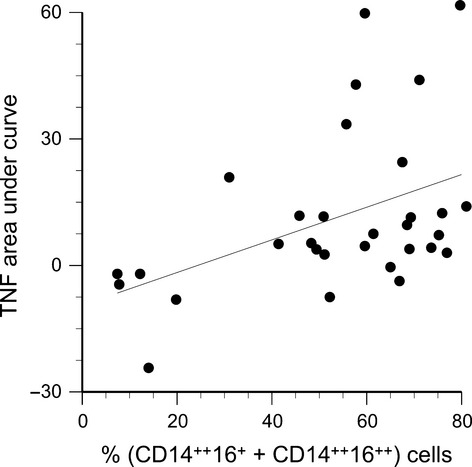
Leptin‐induced TNFα secretion (expressed as area under curve) plotted as function of the percentage of CD14^++^16^+^ and CD14^++^16^++^ monocytes in the blood sample (*R* = 0.44, *P* = 0.01).

**Figure 11.**
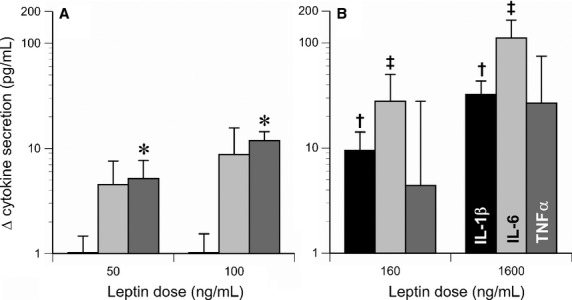
Leptin‐induced increases in IL‐1β, IL‐6 (light gray bars), and TNFα (dark gray bars) in vitro. (A) Responses to physiological doses of leptin by mononuclear cells isolated from the first cohort of subjects (*n* = 34, *P < 0.0001 by repeated measures ANOVA). (B) Responses to supraphysiological doses of leptin by mononuclear cells isolated from the second cohort of subjects (*n* = 11, ^†^*P* = 0.003, ^‡^*P* = 0.025 by repeated measures ANOVA).

Overall, recombinant human leptin induced secretion of TNFα secretion in a dose‐related manner, from baseline levels of 49 ± 11 pg/mL, to 54 ± 12 pg/mL at a dose of 50 ng/mL leptin, and 61 ± 12 pg/mL at 100 ng/mL leptin (*P* < 0.0001). Nevertheless, individual responses varied considerably, with cells from a few subjects even exhibiting slight decreases in TNFα secretion in response to leptin. The variations in response (quantified as areas under the dose response curve) were not related to sex, but did correlate with the summed percentages of CD14^++^16^+^ and CD14^++^16^++^ in the cell cultures (*R* = 0.44, *P* = 0.01, Fig. 10). No significant leptin‐induced changes in IL‐1β or IL‐6 secretion were observed at 50 or 100 ng/mL, however, with the higher doses used with the second cohort of subjects (10 nmol/L [160 ng/mL] or 100 nmol/L [1600 ng/mL]), significant dose‐related increases in IL‐1*β* and IL‐6 were observed (Fig. 11).

### CD14^+^16^++^ monocytes and cardiovascular parameters

**Figure 12.**
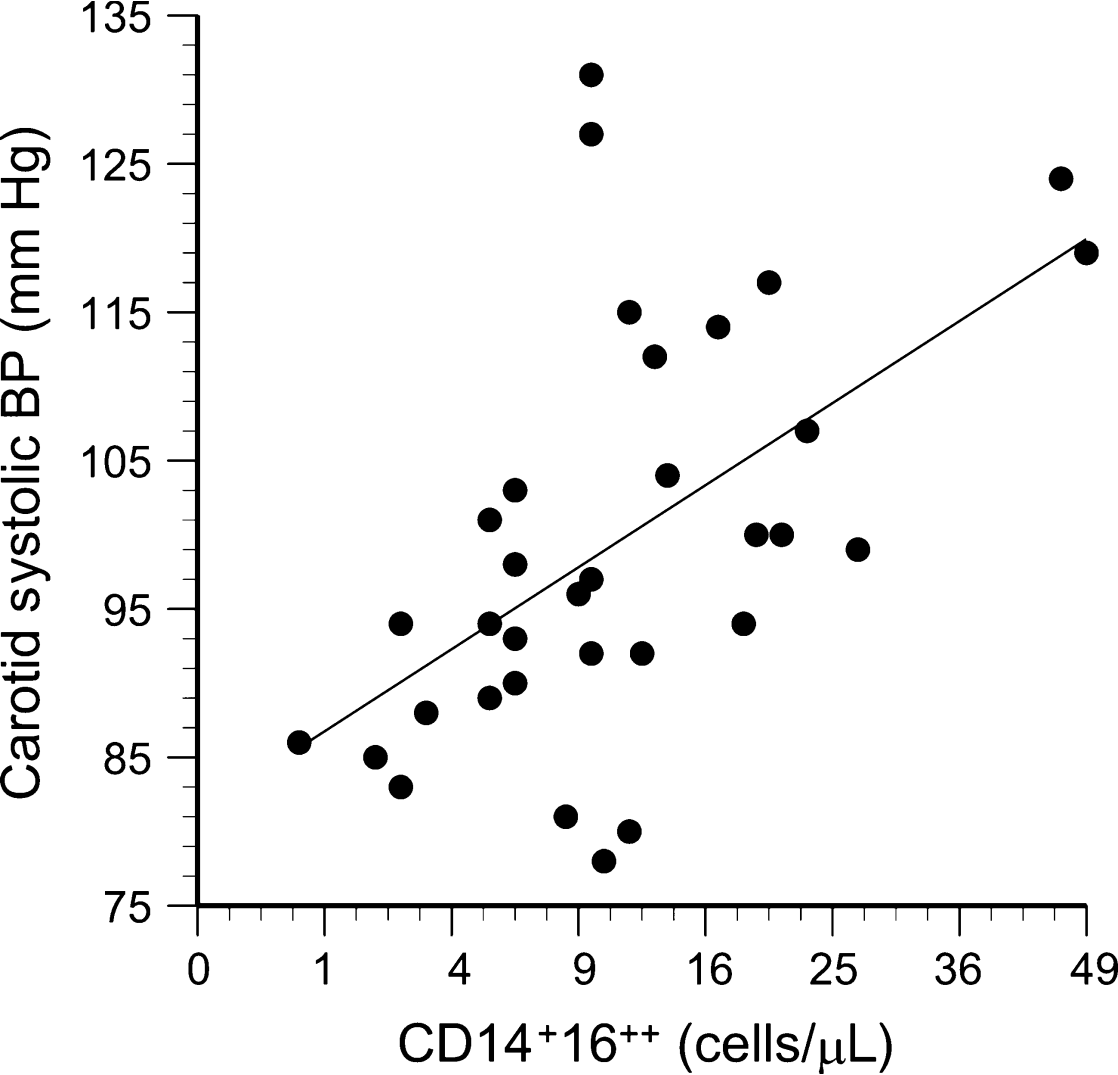
Carotid systolic blood pressure plotted as a function of the number of circulating CD14^+^16^++^ cells (*R* = 0.58, *P* = 0.0008).

**Figure 13.**
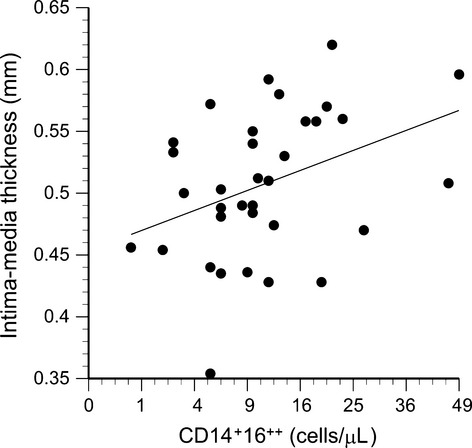
Carotid intima‐media thickness plotted as a function of the number of circulating CD14^+^16^++^ cells (*R* = 0.37, *P* = 0.03).

**Figure 14.**
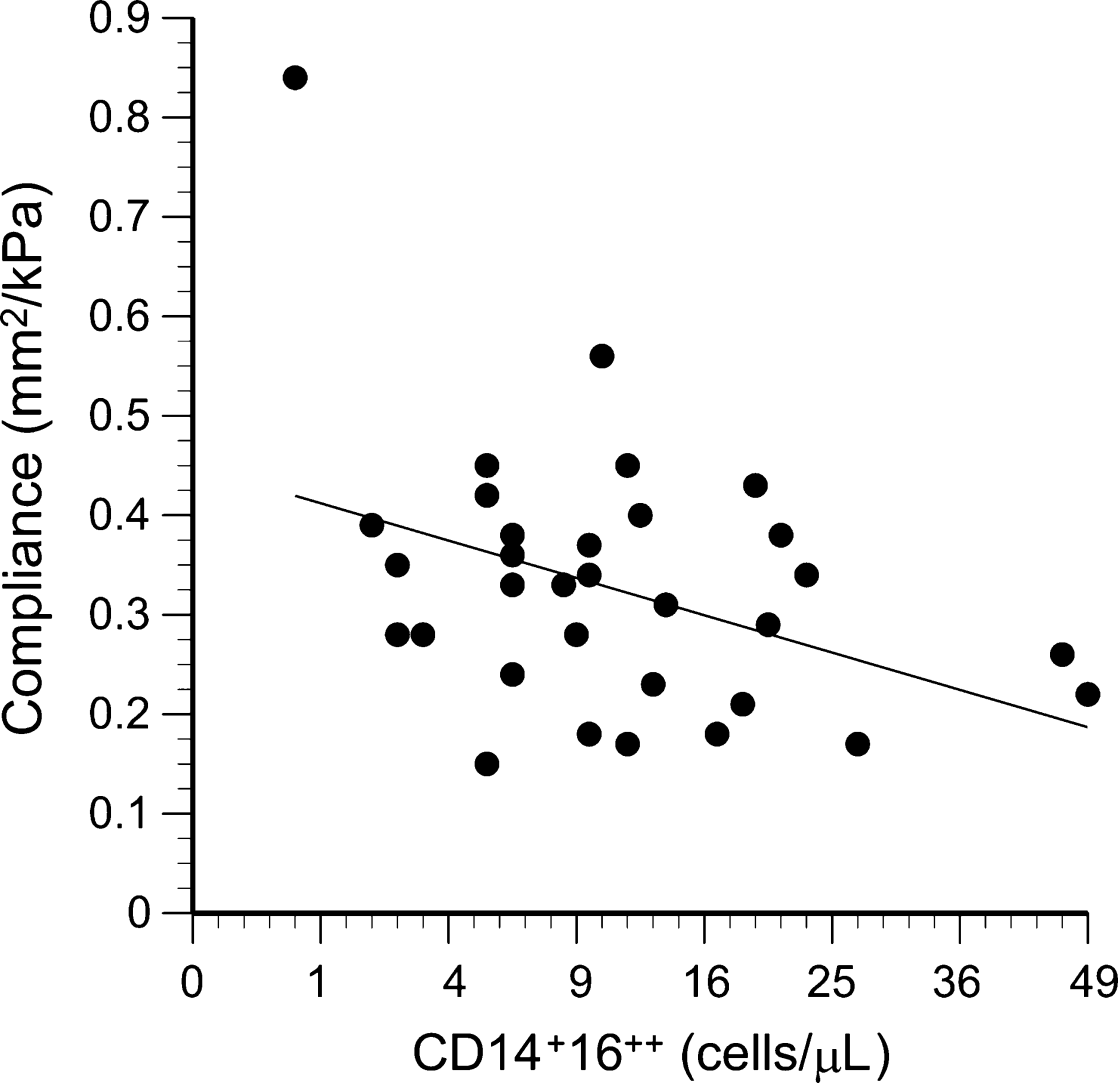
Carotid compliance plotted as a function of the number of circulating CD14^+^16^++^ cells (*R* = −0.39, *P* = 0.02).

Significant correlations were observed between circulating 14^+^16^++^ monocyte counts and carotid systolic blood pressure (*R* = 0.56, *P* = 0.0008, Fig. 12), carotid IMT (*R* = 0.37, *P* = 0.03, Fig. 13), and carotid compliance (*R* = −0.39, *P* = 0.02, Fig. 14). Age, sex, sex hormones, body fat, and traditional cardiovascular risk factors were not retained as significant factors when included in stepwise multiple regressions.

## Discussion

This study provides in vivo (Fig. 4) and in vitro (Fig. 6) evidence that leptin promotes CD16 expression on human monocytes. Furthermore, the present study provides data consistent with previous clinical data (described in the introduction) that CD16‐positive monocytes are associated with diminished vascular function (Figs. 12–14). Thus, on a systemic level, leptin may be a critical link between increased adiposity and vascular disease. The percent body fat in our study population ranged from 10.8% to 43.3%, yet neither body fat nor serum lipoprotein concentrations were significant factors in the multiple regression analyses. This suggests that leptin itself, rather than adiposity or other related factor, is the key element.

This study did not address how leptin may affect CD16 expression on a cellular level. However, it is known that leptin can induce the transcription factors PU.1 (Jaedicke et al. 2013), AP‐1 (Maingrette and Renier 2003), and NF*κ*B (Haugen and Drevon 2007), and that the CD16 promoter has response elements for these transcription factors (Gessner et al. 1995), so a direct effect on CD16 transcription is a possibility.

The present observation that high concentrations of leptin increase CD16 expression on monocytes isolated from the peripheral circulation is consistent with previous gene profiling studies (Ancuta et al. 2009; Wong et al. 2011) and adoptive‐transfer experiments (Yrlid et al. 2006) in supporting a “linear differentiation model.” In this model, monocytes are released from the bone marrow as CD14^++^/CD16‐negative cells, then progress through intermediate stages characterized by strong expression of both CD14 and CD16, and subsequently mature to an advanced stage with diminished expression of CD14. However, the present data do not exclude the alternate possibility that some CD16‐positive monocytes might also originate independently from separate bone marrow precursor cells (Hanna et al. 2011), and this might be the stage that physiological concentrations of leptin have the greatest effect.

Many previous reports have grouped CD14^++^ and CD14^++^16^+^ cells (regions B and C in Fig. 1) together as a single “CD16‐negative” population. As shown in Figure 3, these cells do exhibit similar expression of chemokine receptors. However, in the present study, it was important to differentiate between these regions in order to investigate the influence of leptin on CD16 expression. In fact, in vitro treatment with recombinant leptin produced the greatest increase in region C (CD14^++^16^+^ cells). Thus, differences in endogenous leptin concentrations may explain the variations in circulating CD14^++^16^+^ cells observed among individuals (Fig. 1A vs. B). Moreover, the percentage of CD14^++^16^+^ cells in culture (along with CD14^++^16^++^ cells) correlated with TNFα secretion in vitro (Fig. 10). This latter observation is in accordance with the data reported by Cros et al. (2010), who defined a “CD14^+^16^+^” monocyte subpopulation that corresponded to regions C and D in Figure 1. These CD14^+^16^+^ were the primary producers of IL‐1β and TNFα.

### Sex‐related differences

In our study, circulating CD14^++^16^++^ monocyte counts were higher – and the association with serum leptin was more pronounced – in men than in women. Likewise, recombinant leptin induced CD16 expression on cells isolated from men, but not women. These differences could not be explained by any sex‐related differences in total leptin receptor expression on the monocytes, assessed by flow cytometry. However, flow cytometry only detects the extracellular domains of the leptin receptors and does not differentiate between the long form of the receptor (which has a more extensive cytosolic domain) and several shorter forms with less cytosolic structure and fewer signaling capabilities (Bjorbaek et al. 1997). Western blots were performed to differentiate between receptor isoforms, but no significant sex‐related difference in the relative expression of the long versus short forms was observed. These results are in accordance with two previous studies that found no sex‐related differences in mRNA transcripts for the receptor isoforms in peripheral blood mononuclear cells (Tsiotra et al. 2000; Stefanou et al. 2005). Nevertheless, the sex‐related differences in CD16 expression observed in the present study are consistent with previous reports that women have lower circulating CD16‐positive monocyte counts than men (Heimbeck et al. 2010). Kramer et al. (2004) reported that estradiol inhibited CD16 expression on monocytes in vitro, via mechanisms involving both α and β forms of the estrogen receptor (Kramer et al. 2007). In our study, neither total number of circulating monocytes nor monocyte subpopulations were related to testosterone, estradiol, progesterone, luteinizing hormone, or follicle‐stimulating hormone concentrations.

### Soluble leptin receptor concentrations

The biological activity of leptin can be influenced by soluble leptin receptors that are shed from cell membranes and circulate in the bloodstream (Maamra et al. 2001). At low ratios of circulating soluble leptin receptor‐to‐leptin, the soluble receptors may act as chaperones that extend leptin's biological half‐life, and when present in molar excess (>2/1) they inhibit leptin activity (Huang et al. 2001; Zastrow et al. 2003). In the present study, the median molar ratio was low: 0.74 (interquartile range, 0.86). Serum soluble leptin receptor concentrations were not related to sex, leptin receptor expression on the total monocyte population or to receptor expression on any of the monocyte subpopulations. None of the monocyte‐ or vascular‐related variables measured in this study were associated with soluble leptin receptor concentrations or with soluble receptor/leptin ratios.

### Physical activity and leptin receptor expression

Expression of leptin receptors on CD14^++^ and CD14^++^16^+^ monocytes was negatively correlated with aerobic capacity (Fig. 9). A similar negative influence of physical activity has been observed on leptin receptor expression in other tissues. Hypothalamic tissue from rats given access to exercise wheels for 12 weeks exhibited lower leptin receptor gene expression than tissue from sedentary controls (Kimura et al. 2004). Likewise, leptin receptor gene expression was reduced in the livers of treadmill‐trained rats (Yasari et al. 2009), and in the subcutaneous fat of rats given access to exercise wheels (Pardo et al. 2010). However, physical activity has the opposite influence on leptin receptor expression in skeletal muscle: Wheel running increased leptin receptor protein expression in gastrocnemius and soleus muscles of rats (Pardo et al. 2010), and increased leptin receptor protein was detected in hypertrophied triceps muscles of humans (Olmedillas et al. 2010). The authors suggested that this upregulation represents a local adaptation in leptin signaling that may contribute to training‐induced increases in skeletal muscle fatty acid oxidation.

### CD14^+^16^++^ monocytes and vascular function

In the present study, blood pressure and carotid IMT correlated positively with the number of circulating CD14^+^16^++^. These data are consistent with previous reports that CD14^+^16^++^ monocyte counts correlate with IMT in healthy adults (Rogacev et al. 2010), and with blood pressure in patients with coronary artery disease (Hristov et al. 2010). Prospective studies of patients with chronic kidney disease indicated that elevated CD14^++^16^++^ monocyte counts were associated with higher rates of cardiovascular events in nondialysis patients (Rogacev et al. 2011), and associated with higher rates of both cardiovascular events and fatalities in patients who were on dialysis (Heine et al. 2008).

In the present study, the number of CD14^+^16^++^ monocytes was inversely related to aerobic capacity (Fig. 5). Timmerman et al. (2008) first demonstrated that physically active seniors (65–80 years of age) had lower percentages of circulating CD16‐positive monocytes than sedentary seniors . These authors suggested that exercise‐induced glucocorticoids may cause the effect because methylprednisone therapy reduced CD16‐positive monocyte counts in patients with multiple sclerosis (Fingerle‐Rowson et al. 1998) and in healthy volunteers (Steppich et al. 2000). We did not collect mid‐ or postexercise blood samples to test this possibility, but no significant correlations were observed between resting serum cortisol concentrations and CD14^+^16^++^ monocyte counts. An alternative explanation may be provided by observations that acute exercise causes an immediate increase in CD16‐positive monocyte counts (Steppich et al. 2000), but within an hour of recovery, these counts drop to less than half of pre‐exercise baseline (Simpson et al. 2009). Perhaps regular exercise repeatedly mobilizes marginated CD16‐positive monocytes into the circulation and then they extravasate into muscle and other peripheral tissues that experience low‐level inflammation as a result of the stress of exercise (Cannon et al. 1989), resulting in lower steady‐state numbers in the vascular compartment at rest. The results of the present study suggest a third possibility: Physical activity reduces leptin receptor expression on CD14^++^ monocytes rendering them less susceptible to leptin‐induced CD16 expression.

### Limitations

We began testing the first cohort of subjects before Heimbeck et al. (2010) and Zawada et al. (2012) published important papers illustrating the value of gating the total monocyte population using a pan‐monocyte marker such as CD86 or HLA‐DR, rather than the traditional method of forward‐/side‐scatter characteristics. Selecting cells by scatter characteristics alone can increase the risk of including CD16‐positive neutrophils or NK cells in CD14^+^16^++^ monocyte subpopulation (region E in Fig. 1). The second cohort of subjects provided an opportunity to compare our whole‐blood flow cytometry measurements based on forward‐/side‐scatter gating with CD86 gating. No significant difference in the absolute number of cells measured in region E of Figure 1 was observed, but CD86 gating did eliminate of most of the cells in regions A and F. As a result, enumeration of each monocyte subpopulation as a percentage of total monocytes was underestimated when forward‐/side‐scatter gating was used, compared to CD86 gating. Nevertheless, percentages calculated from the results of each gating method were strongly correlated (e.g., *R* = 0.94, *P* = 0.0002 for the CD14^+^16^++^ subpopulation). Therefore, the relative differences in monocyte subpopulations reported here with respect to sex, leptin, and aerobic capacity would be similar with either gating method.

The effective doses of recombinant leptin required to significantly increase CD16 expression in vitro were higher than the physiological range. This may have been due to an inability to accurately reproduce in vivo conditions in the primary cell cultures. Perhaps endogenous hormone or cytokine cofactors were absent. Alternatively, the timing of leptin administration with respect to monocyte development and/or the duration of incubation may not have been optimal.

Peripheral blood mononuclear cells were analyzed by western blots for differentiation of long and short isoforms of the leptin receptor. Although monocytes express higher levels of leptin receptors than lymphocytes (Zarkesh‐Esfahani et al. 2001), lymphocytes comprise the majority of cells isolated by density gradient centrifugation. If a sex difference in leptin receptor expression was limited to the monocytes or a subpopulation of monocytes, the difference might have been missed due to the presence of lymphocytes.

### Summary

Increased numbers of circulating monocytes expressing CD16 have been associated clinically with increased risk of cardiovascular disease. The present study has shown that expression of CD16 on monocytes correlated with serum leptin concentrations in vivo, and that CD16 expression on monocytes can be induced with recombinant leptin in vitro in a sex‐specific manner. In addition, circulating CD14^+^16^++^ monocyte counts were negatively related to carotid compliance, and positively related to blood pressure and carotid IMT. Finally, leptin receptor expression on CD14^++^ and CD14^++^16^+^ monocytes was negatively correlated with aerobic capacity. Taken together, these observations support the general concept that fatness promotes cardiovascular dysfunction, in part, by leptin‐induced changes in monocyte phenotype, and that fitness opposes this effect by reducing leptin receptor expression on the monocytes.

## Conflict of Interest

None declared.
